# Pneumonia at Base Hospital No. 53, A. E. F.

**Published:** 1919

**Authors:** 


***Pneumonia at Base Hospital No. 53, A. E. F.*** By Major M. L. Goodkind, M. C., Chief of the Medical Service.

The intense concentration of large bodies of men provides suitable conditions for the development of infectious diseases, of which the following are to be considered :

1. Typhoid group : Typhoid, Paratyphoid and Colon.
2. Dysentery - Amebic and Bacillary.
3. The Pneumonias.

The typhoid group is to-day only occasionally met with ; dysenteric disorders are frequent; and it has been concluded that the exciting organism of this group was probably a filtrable parasite, which has not yet been identified.

The lobular type of pneumonia seems to be prevalent in the base hospitals of the A.E.F. The important factor accounting for this fact is the] general frequency of measles and influenza. Another contributing cause is the inhalation of deleterious gases. In the large series of patients admitted to Base Hospital 53, a great proportion of the cases of pneumonia associated with dyspnea or cyanosis, and a great tendency to the development of pulmonary edema were due to the inhalation of mustard, chlorine and phosgene gases.

After eliminating all other causes, influenza still remains as the most potent cause of pneumonia, and it may be considered as responsible for 90 o/o of all the cases.

Bronchopneumonia, which is occasionally designated as lobular or catarrhal, constituted fully 85 o/o of the cases observed. The most frequent factor predisposing to this occurrence was influenza. The physical signs occurred most frequently in the right lung and involved the lower lobe. Occasionally there were marked signs of consolidation, and at times all 3 lobes of the right lung were involved with no signs present in the left lung.

The examination of the blood was fairly uniform in regard to the absence of a leucocytosis. No organism was ever isolated from the blood.

Urinary examination of the influenza patients with probable complication of pneumonia revealed no abnormalities.

At the beginning of the epidemic of influenza the mortality in bronchopneumonia was approximately 35 o/o. From the middle of October to November 25th, empyemas were frequent and pericardial involvements were found in a few instances. In the latter series of cases running a more favorable course, empyema was a fairly frequent complication occurring in approximately 15 o/o of the total number. The physical signs were often misleading.

In the majority of the cases observed, pure culture of pneumococci were obtained from the pus. In the most severely ill, the streptococcus hemolyticus was often found in association. In conjunction with the empyemas, there were a few cases of pneumothorax. There were also cases with marked cerebral disturbances, such as varying degrees of neck rigidity, tendency to Kernig and Brudzinsky, etc., suggesting a possible involvement of the cerebral meninges. The gastro-intestinal tract presented phenomena in a large number of series : intractable emesis associated with diarrhea, etc.

Treatment consisted primarily in the institution of rigid prophylactic measures; living conditions received special consideration. Direct treatment consisted in the early administration of a selected preparation of digitalis, then an interval of rest, etc. Other measures consisted in instituting an appropriate dietetic regime, drinking sufficient fluids, correcting gastro-intestinal disturbances, and in the free administration of oxygen and fresh air. In a certain number of Type 1 patients, anti-pneumococcus serum was employed, but the value of the procedure has not, so far, been ascertained.

The empyema patients were treated conservatively by draining the pus by simple aspiration at intervals of several days, then rib resection was performed.

When signs of cardiac incompetency became apparent, venesection afforded relief. Sterile camphor oil and intravenous injections of adrenalin were occasionally found of value.

